# Phosphodiesterase Type 4D Gene Polymorphism: Association with the Response to Short-Acting Bronchodilators in Paediatric Asthma Patients

**DOI:** 10.1155/2011/301695

**Published:** 2011-08-24

**Authors:** Malgorzata Labuda, Sophie Laberge, Julie Brière, Denis Bérubé, Patrick Beaulieu, Tomi Pastinen, Maja Krajinovic

**Affiliations:** ^1^Research Center, CHU Sainte-Justine, Montreal, QC, Canada H3T 1C5; ^2^Department of Paediatrics, University of Montreal, Montreal, Qc, Canada H3T 1C5; ^3^Department of Human Genetics, McGill University and Génome Québec Innovation Centre, Montreal, Qc, Canada H3A 1A4; ^4^Department of Pharmacology, University of Montreal, Montreal, Qc, Canada H3T 1J4

## Abstract

Short-acting b2-adrenergic receptor agonists are commonly used bronchodilators for symptom relief in asthmatics. The aim of this study was to test whether genetic variants in *PDE4D* gene, a key regulator of b2-adrenoceptor-induced cAMP turnover in airway smooth muscle cells, affect the response to short-acting b2-agonists. Bronchodilator responsiveness was assessed in 133 asthmatic children by % change in baseline forced expiratory volume in one second (FEV_1_) after administration of albuterol. The analyses were performed in patients with airway obstruction (FEV_1_/FVC ratio below 90%, *n* = 93). FEV_1_  % change adjusted for baseline FEV_1_ values was significantly different between genotypes of rs1544791 G/A polymorphism (*P* = 0.006) and −1345 C/T (rs1504982) promoter variation (*P* = 0.03). The association remained significant with inclusion of age, sex, atopy, and controller medication into multivariate model (*P* = 0.004 and *P* = 0.02, resp.). Our work identifies new genetic variants implicated in modulation of asthma treatment, one of them (rs1544791) previously associated with asthma phenotype.

## 1. Introduction

Asthma (MIM 600807) is a common chronic respiratory disease resulting from the complex interaction of genetics and environmental factors [1]. Short-acting b2-adrenergic receptor agonists are commonly used bronchodilators for symptom relief in asthmatics. Activated b2-adrenergic receptor (ADRB2) stimulates adenylyl cyclase leading to increased cyclic adenosine monophosphate (cAMP) level. A primary function of cAMP is to activate protein kinase A (PKA), which in turn activates myosin light chain phosphatase, reducing phosphorylation and generating relaxation [2]. cAMP-activated PKA also activates and phosphorylates phosphodiesterase 4 (PDE4) isoforms which then degrade cAMP providing thus a pivotal acute feedback mechanism [2–4]. 

**Figure 1:**
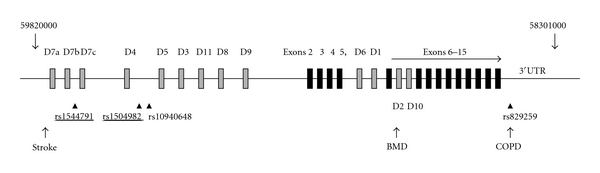
Schematic representation of PDE4D gene. Modified from Houslay [7]. Chromosomal positions are marked at the beginning and at the end of the gene. The orientation of the gene is inversed since PDE4D is encoded on the reverse strand. Common exons are represented by black squares. There are long, short, or supershort splice variants depending on the location of their unique N-terminal regions (D1–D11, grey squares). For simplicity and due to the large size of the gene (over 1.5 Mb), the exon positions are not to scale. Four SNPs used in the present studies are shown, and these significantly associated with b2-response are underlined. Position of SNPs associated with stroke [15], bone mineral density (BMD) [16] and chronic obstructive pulmonary disease (COPD) [17] is indicated. rs1544791 was previously associated with asthma [18].

PDE enzymes, ubiquitously distributed in mammalian tissues, are essential to control numerous physiological processes. PDEs are encoded by 11 gene families, each encompassing 1 to 4 subfamilies of distinct genes [5, 6]. The PDE4 family has gained interest in view of their involvement in the regulation of processes such as inflammation and cognition [3, 7]. Four genes (4A–D) encode a large number of PDE4 isoforms through activation of different promoters or alternative splicing [3, 7]. The dominant PDE4 type expressed in airway smooth muscle cells is *PDE4D* [8–10]. *PDE4D* encode a variety of long and short isoforms which are summarized in Figure 1. Long *PDE4D5* isoform was shown to be a key regulator of b2-adrenoceptor-induced cAMP turnover in human airway smooth muscle cells [11]. The promoter of *PDE4D5* isoform contains a cAMP response element [8]. Chronic elevation of cAMP could therefore lead to desensitization of the receptor to b2-agonists following long-term exposure. This differs from the acute transient desensitization of the receptor caused by PKA phosphorylation and activation or by agonist-triggered b-arrestin-mediated recruitment to the b2-adrenoreceptor [12, 13]. Experiments using knockout mice for PDE4D gene further supported PDE4D role in a regulation of airway smooth muscles contractility [9, 14].

It is thus not surprising that recent genome-wide association study (GWAS) for asthma identified PDE4D as a highly plausible candidate gene [18]. PDE4D was also associated with mineral bone density [16], chronic obstructive pulmonary disease (COPD) in Japanese population [17], and stroke [15, 19]. PDE4D associations with diverse phenotypes are most likely due to its multiple splicing forms with exons scattered over 1.5 Mb, each regulated in a tissue-and-cell specific manner and involved in multiple signalling processes [3, 7, 20]. 

 As the role of PDE4 enzymes was discovered, the interest in controlling their activity was apparent, as summarized in several reviews [2, 20–23]. PDE4 inhibitors have been also viewed as a promising therapy for the treatment of inflammatory airway disorders [24]. The potentially beneficial action of PDE4 inhibitors has been successfully translated in clinical trials for COPD treatment [22, 25, 26], and their efficacy for asthma control is presently under investigation [27].

No data are yet available on the involvement of this gene in the response to drugs used in asthma treatment. The aim of this study was to test the genetic variants in PDE4D gene as potential predictive factor of the response to a single dose of short-acting b2-agonist.

## 2. Patients and Methods

### 2.1. Patients

Patients (*n* = 133) were recruited from the asthma and outpatient pulmonary clinics at Sainte-Justine Hospital between March 2007 and September 2010. Subjects were eligible if they were Caucasians, aged 5 to 18 years, able to perform an appropriate spirometric maneuver under the supervision of technicians specifically trained in pulmonary function testing of children [28], and meeting standard criteria for a diagnosis of asthma [29]. Subjects were eligible for the recruitment irrespectively of the type of maintenance therapy (inhaled corticosteroids, ICS, or leukotriene modifiers) and irrespectively of disease control and severity. Atopy was defined as presence of physician-diagnosed eczema and/or serum food-specific IgE and/or positive skin test for 1 or more aeroallergens or food antigens [30]. Exclusion criteria were as follows: use of long-acting bronchodilators in the last month, prematurity, a history of significant pulmonary disease other than asthma, and known immunodeficiencies. The study protocol was reviewed and approved by the Sainte-Justine Hospital Ethics Committee, and informed consent was signed by each patient's parent.

To assess bronchodilator responsiveness, four inhalations of albuterol were administered via metered dose inhaler (100 *μ*g/inhalation) with valved holding chamber and mouthpiece (Aerochamber; Invacare Corp, Elyria, Ohio, USA). Spirometry was performed before (pre) and 15 min after (post) receiving albuterol sulfate in accordance with the American Thoracic Society guidelines [28]. Short-acting bronchodilators were withheld 6 hours prior to baseline spirometry. Responsiveness to albuterol was reported as the percent change in forced expiratory volume in one second (FEV_1_) after albuterol administration: 100 × ((FEV_1_(post) − FEV_1_(pre))/FEV_1_(pre)). The equations proposed by Polgar and Promadath [31] were selected for the calculation of predicted and reference values.

### 2.2. Genotyping

DNA was extracted from saliva using Oragen kit according to manufacturer (DNA Genotek, http://www.dnagenotek.com). Four SNPs in PDE4D gene were selected for the analysis. Two polymorphisms, located at positions −1345 (rs1504982) and −984 (rs10940648) relative to the first nucleotide of *PDE4D5* mRNA isoform (AF12073), were the only SNPs available at NCBI data base that reside in the 2 kb region preceding *PDE4D5* transcription start site. Two other PDE4D polymorphisms were selected based on published data: rs1544791, located in the intron 2 of the longest PDE4D7 isoform (NM_001165899.1), was a top-ranking SNP in the GWAS of asthma [18] and the other, rs829259, located in 3′UTR of all PDE4D isoforms, was reported in association with the COPD in adults [17].

All DNA segments containing the polymorphic sites were amplified by PCR in a total volume of 20 ul using 15 ng of genomic DNA, 0.4 *μ*M of each primer, 140 *μ*M dNTPs, 10 mM Tris-HCl (pH 8.3), 2.3 mM MgCl_2_, 50 mM KCl, and 0.4 U of Platinum Taq polymerase (Invitrogen). Amplification was performed for 37 cycles composed of 30 s at 94°C, 30 s at 57°C, and 30 s at 72°C, following initial denaturation of 3 min at 95°C. Genotyping was performed by allele-specific oligonucleotide (ASO) hybridization as described previously [32]. The sequence of primers used for PCR and ASO is provided in Table 1.

### 2.3. Statistical Analysis

The % change in FEV_1_ was analyzed by analysis of covariance (ANCOVA). Genotype was included in the model as fixed factor, and the baseline FEV_1_% predicted value was included as continuous covariate. The analyses were restricted to patients having airway obstruction, for example, baseline FEV_1_/FVC (FVC—forced vital capacity) ratio lower than 90% [33, 34] (see Table 2 for patients characteristics). Hierarchical multivariate linear regression was used to assess the association between genotypes of rs1544791 and rs1504982 with the inclusion of additional covariates (age, sex, atopy, and use or not of ICS and leukotriene modifiers). The analyses were performed by SPSS (Version 17.0).

## 3. Results and Discussion

Details of patients' characteristics are provided in Table 2. Asthma severity of patients recruited in our study is comparable to that observed in the Childhood Asthma Management Program (CAMP) and the Leukotriene Modifier or Corticosteroid or Corticosteroid Salmeterol trial (LOCCS) populations [35] used in a number of pharmacogenetic studies.  Table 3 presents mean % FEV_1_ change according to genotypes in PDE4D gene. Significant differences were obtained for *PDE4D5* promoter −1345 (rs1504982, *P* = 0.03) and for rs1544791 polymorphisms (*P* = 0.006). The mean % FEV_1_ change ± SE for rs1504982 C carriers was 7.1 ± 0.7 versus 10.3 ± 1.2 in noncarriers, and for rs1544791 G carriers, the mean % FEV_1_ change ± SE was 7.4 ± 0.7 as compared to 13.7 ± 2.1 in individuals without this allele. The association remained significant for both SNPs with inclusion of age, sex, atopy, and controller medication into the multivariate model (*P* = 0.02 and *P* = 0.004, resp.). 

The recent association of PDE4D to asthma phenotype [18], as well as its known function, makes PDE4D an excellent candidate gene for the association with the outcome of asthma treatment. PDE4D is a well-established regulator of airway smooth muscles contractility [9, 14] and plays a key role in ADRB2-induced cAMP regulation [11]. Study conducted *in vitro* demonstrated that a single nucleotide substitution in one of cAMP-responsive elements in *PDE4D5* promoter created by site-directed mutagenesis, causes cAMP-driven upregulation of *PDE4D5* expression and functional activity in human airway smooth muscle cells [8]. Based on the publicly available data, it does not seem that there is a naturally occurring genetic variant in *PDE4D5* cAMP-responsive elements. Among SNPs analyzed here, −1345 variation of the *PDE4D5* promoter was associated with the change in FEV_1_ following a single dose of short-acting b2-agonist. The change in FEV_1_ was also significantly associated with rs1544791, the top ranking SNP in GWAS study of asthma susceptibility [18]. The same G asthma predisposing allele was associated in our study with the poor treatment outcome. 

Schematic representation of PDE4D gene structure and relation to PDE4D isoforms is given in Figure 1. SNPs tested within this study are depicted as well as those associated with variety of phenotypes reported by others [15–17]. Each of the phenotypes associated with PDE4D gene (stroke, asthma, BMD, and COPD) maps to different linkage disequilibrium (LD) blocks. The same is true for the SNPs significantly associated with b2-agonist response in this study. The SNP rs1544791 is located within 181 kb intron 2 of the longest isoform (PDE4D7, NM_001165899.1). This SNP resides within 75 kb long LD block and is separated by 374 kb from *PDE4D5* transcription start. Despite its distant location, it could act as a remote cis-acting element. Indeed, functional-SNP database (F-SNP) integrating information from 16 bioinformatics tools [36] predicts that it might be a transcription regulator. We further assessed rs1544791 in allelic expression dataset generated in HapMap lymphoblastoid (CEU) cell lines of European ancestry [37] where allelic differences within individual were renormalized as recently described [38]. Population differences for allelic expression of uc010iwj.1 isoform (also corresponding to the first few exons unique to the longest PDE4D7 isoform NM_001165899.1 [7]) were significantly associated (*P* = 0.005) with rs1544791 genotype. SNP −1345 is also recognized as transcription regulator in F-SNP data base [36] and in UCSC browser (http://genome.ucsc.edu). 

In the analysis, we used % FEV_1_ change as a continuous variable with the correction for baseline FEV_1_% predicted values rather than categorical phenotype of responders and nonresponders based on a % FEV_1_ change above or below a given predefined value. The reason for this was a strong positive correlation between poor response to the administered albuterol and high baseline FEV_1_% predicted values. Since about 50% of our patients were on regular ICS and 20% were on leukotriene modifiers, it is reasonable to assume that prior treatment with these drugs had improved lung function in a proportion of our study subjects, therefore reducing the magnitude of the response to albuterol. Because of ethical considerations, we did not withdraw anti-inflammatory treatment prior to assessment, but we therefore restricted our analysis to the patients with airway obstruction. Since airway obstruction is defined by a reduced FEV1/FVC ratio [34] which normally reaches a value of 0.9 in children [33], we limited our analysis to patients with FEV1/FVC ratio lower than that threshold. An alternative strategy would have been to recruit new asthma patients prior to initiation of any asthma controller therapy. However, onset of childhood asthma usually occurs early during the preschool age and valuable spirometric data cannot be obtained in preschoolers. A multicenter study would have been necessary in order to recruit the appropriate numbers of patients.

## 4. Conclusions

This study demonstrates the first attempt to explore the association of PDE4D genetic variations and response to asthma b2-agonists treatment in Caucasian children. Results obtained are not necessarily applicable to other populations as frequency of tested polymorphisms and LD structure differ [18]. If confirmed in a larger study, this finding will build upon existing knowledge in asthma pharmacogenetics ultimately assisting personalized patient treatment.

##  Funding

The work was supported by a Grant from the Canadian Institutes of Health Research (MOP-81154); M. Krajinvoic is a scholar of the Fonds de la Recherche en Santé du Québec. T. Pastinen is supported by CIHR and holds a CRC (tier 2).

## Figures and Tables

**Table 1:** Primers used for amplification and genotyping of PDE4D polymorphisms.

SNP ID	Position	PCR primers	ASO primers
rs1544791	Intron 2	GAAGCATGTTGCACATAAACTG,	ATGTGGCA/GTTATTTGC
		TCTCTTCTACTCTATGGGCTTTCTG	
rs1504982	−1345	GGGAGCTACAAAATGCATCC,	ATTCCTTGC/TCTTTTAAA
		CAAGTGGTTGACCCTGTCCT	
rs10940648	−984	CGTGATTTCCTTTATTGCCTTT,	AGTAAAAAT/CGTTGTAAC
		TTCCCTATGCAAAGGTTTGAG	
rs829259	3′UTR	TTGTCCCTGAGTGAAGTCTAGAAA,	TCCCTACT/ACTTAGTAT
		CCCTTTCAGGTCTGGATTTG	

rs1544791 is located in intron 2 of PDE4D7 isoform; positions of rs1504982 and rs10940648 refer to the start of *PDE4D5* mRNA (AF012073) and, rs829259 is located in 3′UTR, common to all splice variants.

**Table 2:** Patients' characteristics.

Characteristic*	Total (*n* = 133)	Total (*n* = 93)
Age, years	9.8 (±0.3)	10.3 (±0.3)
Males, *n* (%)	73 (54.9)	55 (59.1)
Females, *n* (%)	60 (45.1)	38 (40.9)
Atopy**, *n* (%)	100 (75.2)	73 (78.5)
Parental smoking, *n* (%)	36 (27.1)	27 (29.0)
Regular ICS use, *n* (%)	68 (51.1)	48 (51.6)
Leukotriene modifier use, *n* (%)	28 (21.1)	21 (22.6)
Baseline FVC, % predicted	106.8 (±1.1)	108.6 (±1.3)
Baseline FEV_1_, % predicted	96.9 (±1.2)	94.0 (±1.2)
Baseline FEV_1/_FVC ratio	84.5 (±0.8)	80.5 (±0.7)

*Patients' characteristics are given as number (%) or mean ± SE for entire group of patients (*n* = 133) or for patients with FEV_1_/FVC ratio below 90% (*n* = 93) to which the genotype analysis was restricted to; **presence of physician-diagnosed eczema, food-specific IgE, and/or positive skin test for 1 or more aeroallergens or food allergens [30]; ICS: inhaled corticosteroids, FVC: forced vital capacity, FEV_1_: forced expiratory volume in one second.

**Table 3:** FEV_1_% change following administration of albuterol according to PDE4D genotypes.

SNP/genotypes	*N*	% FEV_1_ change (mean ± SE)**	P***
**rs1544791 intron 2**			
AA	8	13.7 (±2.1)	**0.01**
AG	34	6.6 (±1.0)	
GG	51	7.9 (±0.8)	
**GG + GA versus AA**		**7.4 ± 0.7 versus 13.7 ± 2.1**	**0.006**

rs1504982 (−1345*)			
CC	22	7.0 (±1.3)	0.08
CT	46	7.1 (±0.9)	
TT	25	10.3 (±1.2)	
**CC + CT versus TT**		**7.1 ± 0.7 versus 10.3 ± 1.2**	**0.03**

rs10940648 (−984*)			
CC	6	8.2 (±2.6)	0.4
TC	28	6.6 (±1.2)	
TT	59	8.5 (±0.8)	

**rs829259 **			
AA	13	8.5 (±1.7)	0.1
AT	45	6.6 (±0.9)	
TT	35	9.5 (±1.1)	

rs1544791 is located in intron 2 of PDE4D7 isoform; *positions of rs1504982 and rs10940648 refer to the start of *PDE4D5* mRNA (AF012073), and rs829259 is located in 3′UTR common to all splice variants; **mean values adjusted for baseline FEV_1_; ***P derived from ANCOVA.
